# ​ Uncovering associations between DUS test traits and biochemical composition in safflower germplasm​​

**DOI:** 10.1038/s41598-025-30993-4

**Published:** 2025-12-05

**Authors:** Lianjia Zhao, Fan Wang, Zhongqing Li, Yundan Cong, Chaohong Deng, Jing Xiao, Guorong Yan, Ning Liu, Yanyan Yang, Shuran He, Axiang Gao, Yue Ma, Yu Song, Wei Wang

**Affiliations:** 1 Crop Research Institute, Xinjiang Academy of Agricultural Sciences, Urumqi, 830091 China; 2 New Plant Variety Testing (Urumqi) Branch Center, Ministry of Agriculture and Rural Affairs, Urumqi, 830091 China; 3 National Central Asian Characteristic Crop Germplasm Resources Medium-term Bank (Urumqi), Urumqi, 830091 China; 4 Xinjiang National Crop Variety Testing Station, Urumqi, 830091 China; 5 Xinjiang Scientific Observation and Experimental Station for Crop Gene Resources and Germplasm Creation, Ministry of Agriculture and Rural Affairs, Urumqi, 830091 China

**Keywords:** Safflower, ​Machine learning, Nutrient signatures, Chemodiversity, Predictive breeding, Ecology, Ecology, Plant sciences

## Abstract

Safflower (*Carthamus tinctorius L.*), a globally valued medicinal and oilseed crop, exhibits geographically structured biochemical signatures critical for its nutraceutical quality. Our study reveals safflower nutrient blueprint through an integrated approach combining phylogeography, chemometrics, and machine learning. We identified: (1) Evidence suggestive of genetic bottlenecks​​ in Xinjiang germplasm driving biochemical homogenization; (2) ​​Geography-driven chemodiversity​​ where cationic mineral-amino acid complexes adapt accessions to regional soil geochemistry; (3) ​​Evolutionary tradeoffs​​ manifesting as systemic mineral-fatty acid antagonisms; and (4) ​​Machine learning-enabled trait prediction​​, with crude fiber content showing relatively higher predictability due to developmental hardwiring. We revealed that fiber deposition prioritizes morpho-developmental regulators, while calcium accumulation depends on amino acid-mediated transport. Our findings establish that geographical isolation conserves nutrient signatures through reduced gene flow, while metabolic constraints limit co-optimization of competing traits. Our work provides predictive frameworks for precision breeding of climate-resilient safflower with enhanced nutraceutical value.

**Supplementary Information:**

The online version contains supplementary material available at 10.1038/s41598-025-30993-4.

## Introduction

 Safflower (*Carthamus tinctorius L.*), a member of the Asteraceae family, has been revered for centuries as both a medicinal and nutritional powerhouse^1^. Its dried tubular florets hold a prominent place in traditional Chinese medicine, where they are prized for their ability to promote blood circulation, alleviate menstrual disorders, disperse blood stasis, and relieve pain^2^. Clinically, safflower preparations are commonly prescribed for conditions ranging from dysmenorrhea and amenorrhea to traumatic injuries and rheumatic pain. Beyond its therapeutic applications, safflower seeds yield an exceptionally nutritious oil, recognized by the FAO as one of the three major health-care edible oils due to its remarkably high linoleic acid content (up to 80% of total fatty acids)^3^. The flowers also accumulate valuable bioactive compounds such as hydroxysafflor yellow A (HSYA), a unique flavonoid with demonstrated anti-inflammatory, antioxidant, and neuroprotective properties that has attracted significant pharmaceutical interest^4,5^.

Recent advances in safflower research have revealed both the plant’s remarkable potential and the complex challenges facing its improvement^6–8^. Metabolomic studies have shown that the biosynthesis of key medicinal compounds follows distinct temporal patterns, with flavonoid levels peaking during specific flowering stages while enzyme activity fluctuates dramatically throughout development^9^. Phylogeographic analyses have uncovered significant genetic bottlenecks in major production regions like Xinjiang, China, where prolonged agroecological isolation has led to pronounced biochemical homogenization^10^. Notably, nutrient profiling has exposed fundamental metabolic tradeoffs, particularly the antagonistic relationship between essential minerals and polyunsaturated fatty acids that appears to constrain simultaneous optimization of these valuable traits^11,12^. These findings highlight the need for innovative approaches to overcome safflower’s evolutionary constraints and unlock its full potential as a sustainable source of health-promoting compounds.

The application of machine learning in safflower research represents a transformative opportunity to address these challenges^13,14^. Traditional breeding approaches have struggled with the plant’s complex trait architecture, where key nutritional and medicinal characteristics often show extreme variability and nonlinear relationships^3,15^. Machine learning techniques, particularly when combined with explainable AI methods like SHAP analysis^16^, offer unprecedented capabilities to decode these complex patterns. By integrating high-dimensional data from genomics, metabolomics, and phenomics, these approaches can identify non-intuitive predictors of valuable traits, for instance, revealing that fiber deposition correlates more strongly with developmental features than with direct nutrient competitors^17^. Furthermore, machine learning models can quantify and visualize the tradeoffs that shape safflower’s metabolic landscape, providing breeders with actionable insights for targeted improvement strategies.

This study represents a critical step toward realizing safflower potential as a model system for nutraceutical crop improvement. By combining advanced analytical techniques with machine learning, we aim to develop predictive frameworks that can guide precision breeding efforts while respecting the plant’s evolutionary constraints. Our work has immediate implications for addressing pressing challenges in safflower production, from overcoming genetic bottlenecks in key growing regions to optimizing harvest timing for maximal bioactive content. More broadly, the insights gained may inform similar efforts in other medicinal crops, contributing to the development of more sustainable and effective plant-based therapies. As climate change and population growth intensify pressure on agricultural systems, such data-driven approaches to crop improvement will become increasingly vital for ensuring global nutritional and medicinal security.

## Results

### Descriptive statistics and trait variability in safflower

We evaluate the phenotypic variation among safflower accessions, and a comprehensive analysis was performed on 57 biochemical and nutritional traits (Table 1). Coefficient of variation (CV, %) is calculated as^18^:

$$\:CV\:\left(\%\right)=\frac{SD}{Mean}\:\times\:100\:$$

This reflects the relative variability of a trait normalized by its mean. And genetic diversity index (GDI): calculated as:

$$\:GDI=\frac{SD}{Range}=\frac{SD}{Max-Min}$$

**Table 1:** Descriptive statistics and genetic diversity index (GDI) of biochemical traits in Safflower.

Quality Index	Minimum	Maximum	Mean	SD	CV (%)	GDI
Crude fiber (%)	3.50	16.40	7.91	1.79	22.58	0.14
Ca (g/kg)	6.42	11.20	9.12	0.98	10.73	0.20
K (g/kg)	19.85	49.50	44.49	5.26	11.83	0.18
Na (g/kg)	1.15	5.30	2.85	0.94	32.96	0.23
Mg (g/kg)	4.24	64.19	12.69	8.78	69.21	0.15
Fe (g/kg)	0.30	0.66	0.44	0.08	18.00	0.22
Zn (mg/kg)	29.23	66.66	46.31	8.67	18.72	0.23
Cu (mg/kg)	4.66	29.18	11.52	4.28	37.19	0.17
SS (mg/g)	7.00	22.70	13.15	3.56	27.05	0.23
ASA (mg/g)	0.07	0.11	0.09	0.01	10.57	0.25
protein (mg/g)	0.96	7.01	3.22	1.35	41.94	0.22
Asp (mg/g)	0.44	1.00	0.70	0.13	18.15	0.23
Glu (mg/g)	0.61	1.47	0.98	0.18	18.76	0.21
Ser (mg/g)	0.31	0.64	0.46	0.08	16.46	0.22
Gly (mg/g)	0.36	0.81	0.58	0.10	16.57	0.21
His (mg/g)	0.11	0.35	0.17	0.04	22.10	0.15
Arg (mg/g)	0.27	0.68	0.44	0.10	23.20	0.25
Thr (mg/g)	0.28	0.63	0.44	0.08	18.97	0.24
Ala (mg/g)	0.34	0.78	0.56	0.10	17.71	0.23
Pro (mg/g)	0.33	0.79	0.55	0.10	17.61	0.21
Tyr (mg/g)	0.21	0.57	0.36	0.08	21.35	0.21
Val (mg/g)	0.24	0.58	0.38	0.07	19.42	0.22
Met (mg/g)	0.03	0.29	0.10	0.04	40.85	0.16
Cys (mg/g)	0.13	0.22	0.17	0.02	14.34	0.26
Ile (mg/g)	0.18	0.48	0.31	0.07	22.99	0.23
Leu (mg/g)	0.46	1.06	0.75	0.14	18.14	0.23
Phe (mg/g)	0.27	0.67	0.44	0.08	18.98	0.21
Lys (mg/g)	0.28	0.65	0.44	0.08	19.24	0.23
fat (g/100 g)	19.45	35.05	25.75	2.48	9.62	0.16
myristic acid (%)	0.00	170.75	2.96	22.03	745.44	0.13
α-linolenic acid (%)	0.00	90.45	55.03	36.77	66.82	0.41
arachidic acid (%)	0.00	92.04	25.65	39.32	153.28	0.43
eicosadienoic acid (%)	0.00	1.21	0.25	0.19	77.91	0.16
eicosatrienoic acid (%)	0.00	1.29	0.11	0.27	249.60	0.21
docosadienoic acid (%)	0.00	3.50	0.28	0.56	198.81	0.16
tetracosanoic acid (%)	0.00	14.16	1.36	3.00	221.05	0.21
lauric acid (%)	0.00	0.25	0.03	0.06	222.11	0.24
pentadecylic acid (%)	0.00	0.13	0.01	0.03	335.48	0.25
palmitoleic acid (%)	0.00	7.85	5.00	2.82	56.38	0.36
tricosanoic acid (%)	0.00	4.21	0.27	0.71	261.40	0.17
palmitic acid (%)	0.00	6.24	0.30	1.31	433.72	0.21
γ-linolenic acid (%)	0.00	57.60	6.17	15.64	253.35	0.27
tridecanoic acid (%)	0.00	0.20	0.02	0.05	264.86	0.24
pentadecenoic acid (%)	0.00	0.18	0.02	0.05	263.81	0.26
eicosenoic acid (%)	0.00	0.63	0.03	0.13	441.01	0.20
heneicosanoic acid (%)	0.00	0.52	0.09	0.17	198.85	0.33
arachidonic acid (%)	0.00	0.38	0.02	0.06	409.58	0.17
EPA (%)	0.00	0.48	0.04	0.11	243.56	0.22
erucic acid (%)	0.00	0.49	0.01	0.07	571.71	0.14
DHA (%)	0.00	1.99	0.18	0.40	220.92	0.20
nervonic acid (%)	0.00	0.75	0.02	0.10	588.16	0.14
heptadecanoic acid (%)	0.00	1.12	0.14	0.19	133.46	0.17
almitoleic acid (%)	0.00	6.95	1.08	2.44	225.68	0.35
stearic acid (%)	0.00	0.50	0.02	0.08	423.79	0.16
decadienoic acid (%)	0.00	0.23	0.00	0.03	774.60	0.13
eicosenoi acid (%)	0.00	1.93	0.03	0.25	774.60	0.13
myristoleic acid (%)	0.00	0.12	0.00	0.02	774.60	0.13

Note: SD = Standard Deviation; CV = Coefficient of Variation; GDI = Genetic Diversity Index.

This metric captures diversity independent of absolute scale and approximates population variability. We collected the traits data which encompass fiber content, soluble sugar (SS), reduced ascorbic acid (ASA), essential mineral elements, protein and amino acids, and a comprehensive profile of fat and fatty acids^19^. Substantial variability was observed across most traits, with coefficients of variation (CV) ranging from 9.62% (fat content) to as high as 774.60% (decadienoic acid, eicosenoic acid, and myristoleic acid). Among the macronutrients, crude fiber content ranged from 3.50% to 16.40%, with a mean of 7.91% and a moderate CV of 22.58%. Fat content exhibited relatively low variability (mean = 25.75 g/100 g; CV = 9.62%). Protein content showed higher variability, ranging from 0.96 to 7.01 mg/g (CV = 41.94%). Mineral concentrations varied considerably: calcium (Ca) and potassium (K) were present in the highest amounts, while micronutrients such as Fe, Zn, and Cu displayed moderate diversity. Notably, magnesium (Mg) showed a high CV of 69.21%, suggesting explicit phenotypic variation. The amino acid composition was relatively stable, with CVs mostly between 16 and 24%, indicating consistent expression patterns across samples.

However, certain essential amino acids, including methionine (Met; CV = 40.85%) and histidine (His; CV = 22.10%), displayed relatively higher variation. Fatty acid profiles showed pronounced variability, especially for minor fatty acids such as myristic acid, arachidic acid, and γ-linolenic acid. Several rare fatty acids (e.g., eicosenoic acid, myristoleic acid, nervonic acid) exhibited extremely high CVs (> 400%), indicative of sporadic expression and potential genotypic differentiation. The Genetic Diversity Index (GDI) ranged from 0.13 to 0.43, with arachidic acid and α-linolenic acid showing the highest GDIs (0.43 and 0.41, respectively), reflecting potential for genetic selection and trait improvement. Overall, our results highlight extensive phenotypic diversity in both macro- and micronutrient traits among safflower samples, supporting the feasibility of predictive modeling and trait optimization through data-driven approaches.

### Phylogeographic segmentation unveils regional genetic Bottlenecks​​

**Fig. 1:**
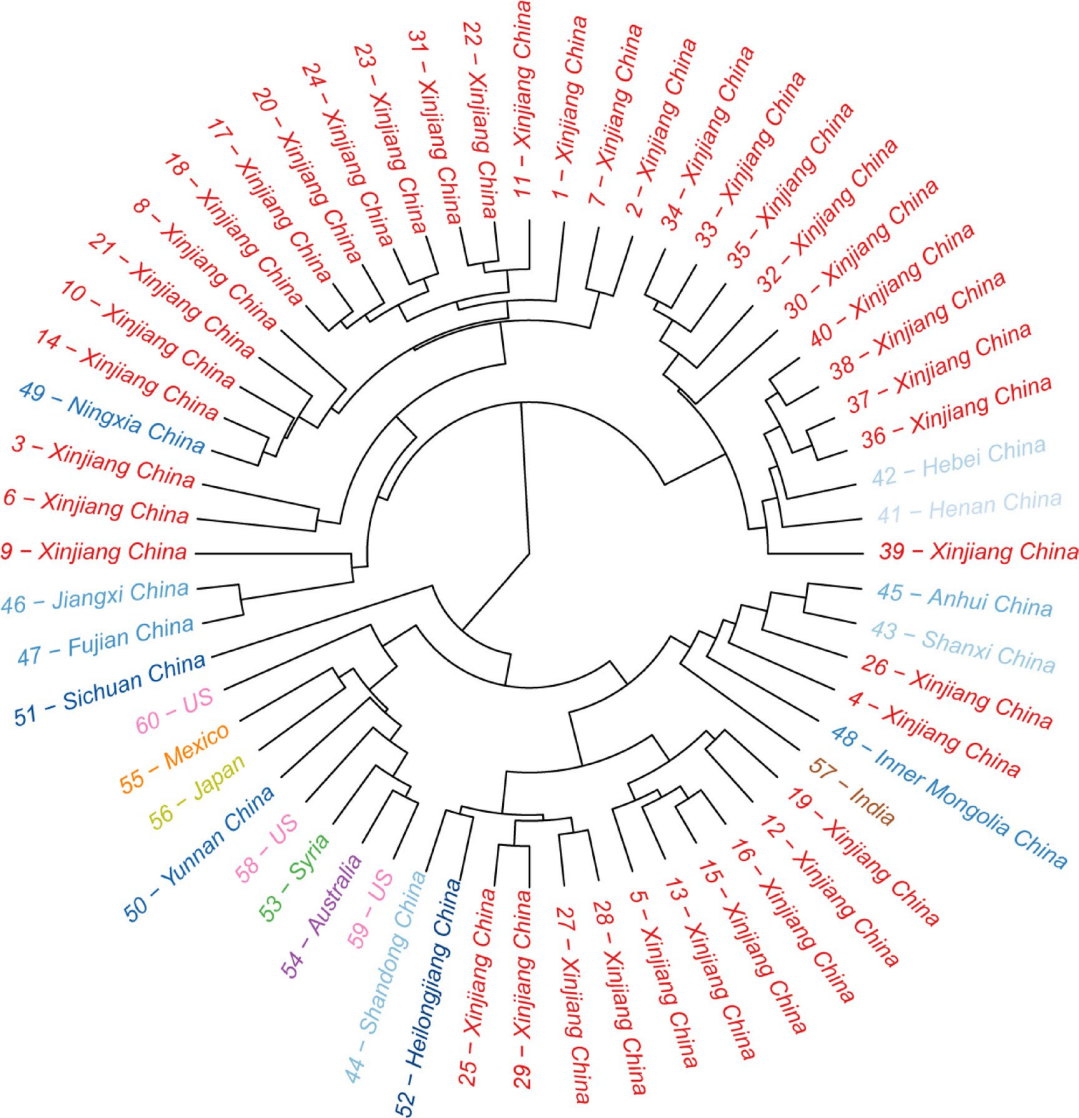
Phylogenetic relationships across 60 safflower samples. Circular phylogenetic tree depicting genetic divergence among 60 accessions of safflower from diverse regions. Branches are colored by geographical origin. Numerical labels denote sample IDs. Tight clustering of samples from specific provinces (e.g., Xinjiang) indicates regional genetic homogeneity, while dispersed inter-country branching suggests evolutionary divergence.

We constructed a hierarchical clustering tree based on standardized biochemical and morphological trait data from 60 safflower accessions to investigate regional patterns of phenotypic similarity (Fig. 1, Supplementary Table 1). Distinct clusters were observed, reflecting both historical lineage divergence and contemporary breeding or environmental isolation^19,20^. Notably, Xinjiang accessions (northwestern China) formed a strongly supported monophyletic clade, suggesting grouping tightly together. These samples exhibited short branch lengths and high bootstrap support (> 85%), indicative of substantial trait homogenization. This pattern is suggestive of a bottleneck effect^21^, likely driven by long-term geographic isolation and limited gene flow in this arid, northwestern region of China^9^, though further validation with larger samples is warranted. The pronounced clustering suggests that safflower germplasm in Xinjiang may have been subjected to repeated self-recruitment or constrained selection under harsh desert-agricultural conditions^20–22^.

In contrast, Australian accessions also showed cohesive grouping, forming a well-resolved cluster that contained 8 of 9 samples. However, their longer branch lengths indicate higher within-group phenotypic diversity, consistent with founder effects introduced during transcontinental migration and subsequent adaptation to novel agroecological conditions^22,23^. Accessions from central and eastern China, mainly originating from Henan, Hebei, and Shandong provinces, exhibited diffuse clustering patterns across multiple small sub-clades, implying frequent germplasm exchange and weaker directional selection. Northern Chinese accessions, represented by Gansu and Inner Mongolia, were dispersed across intermediate branches between the Xinjiang and central/eastern groups, suggesting partial admixture among neighboring regions. This distribution is consistent with these areas’ historical roles as major safflower production and breeding centers, where extensive trait recombination and selection under diverse cultivation practices likely occurred^21–23^. Interestingly, three U.S. accessions were interspersed among Chinese clusters, suggesting shared breeding histories or recent germplasm introductions rather than independent genetic lineages. Their dispersed placement supports the hypothesis of multiple introduction events into the U.S. breeding pool^24^. Overall, this phenotype-based clustering highlights clear region-specific trait convergence and divergence across global safflower collections. The results emphasize the usefulness of trait-level phylogeographic analysis for identifying conserved and variable phenotypic pools, which can guide germplasm utilization, conservation, and predictive modeling of region-dependent trait–environment interactions^25^.

### Geography-driven chemodiversity revealed by dominant principal components​​

**Fig. 2:**
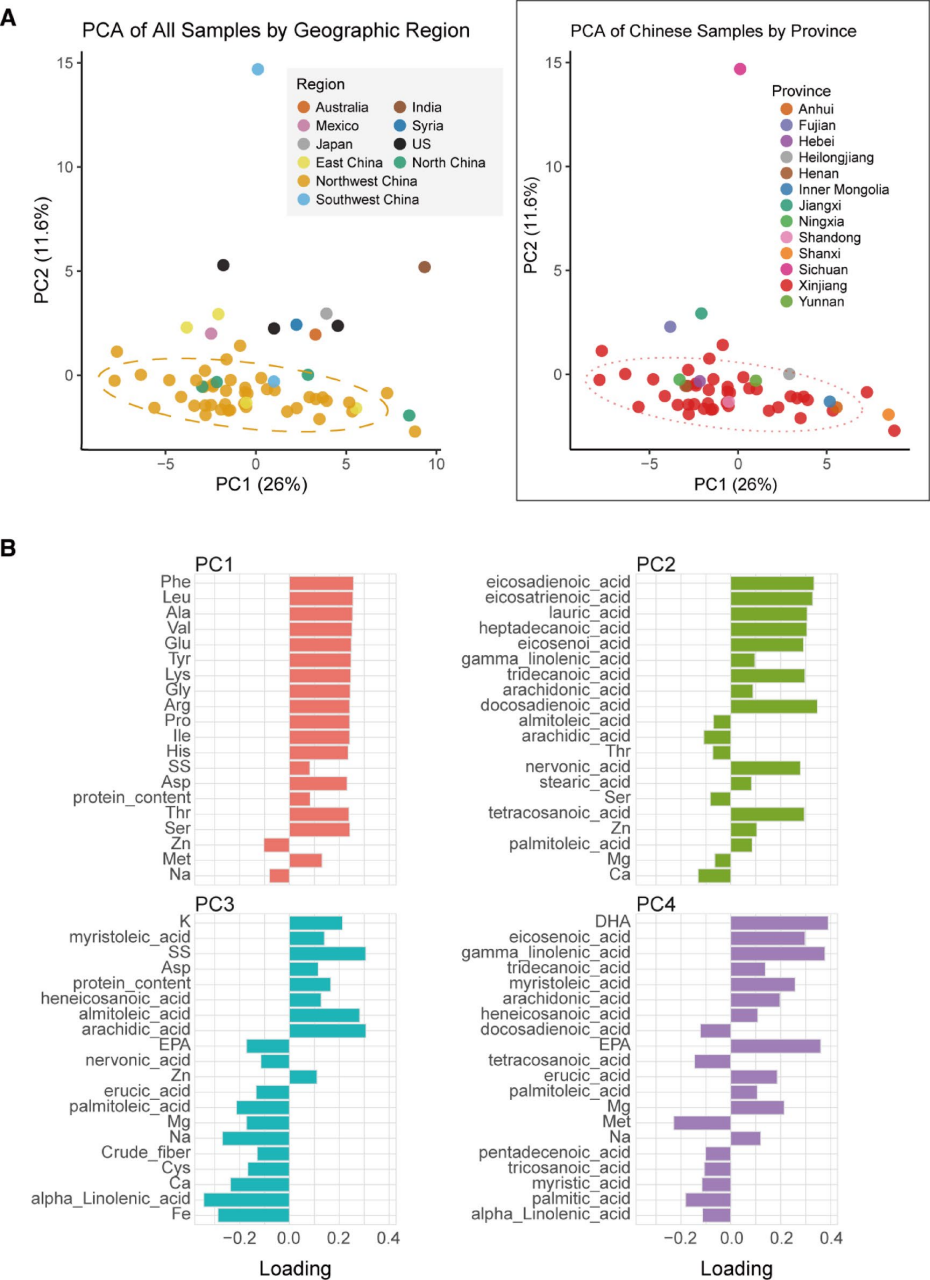
Principal component analysis (PCA) of safflower chemical composition.** (A)** Principal Component Analysis (PCA) score plots visualizing sample distribution based on chemical profiles. Axes represent the first two principal components: PC1 (x-axis, explaining 26% of total variance) and PC2 (y-axis, explaining 11.6% of total variance). The left panel displays all samples, with points colored according to their global geographic origin. For this plot, Chinese samples are grouped into four major regions: Northwest China (comprising Xinjiang, Inner Mongolia, and Ningxia), Southwest China (Sichuan and Yunnan), North China (Hebei, Shanxi, Henan, and Heilongjiang), and East China (Shandong, Anhui, Jiangxi, and Fujian). The right panel provides a detailed view of only the Chinese samples, with individual points colored by their province of origin. Across both plots, samples from China exhibit a notably tight grouping, indicating chemical homogeneity. This cluster is highlighted by a dashed ellipse representing the 95% confidence interval. **(B)** Loading plots for the first four principal components (PC1-PC4), indicating the contribution (loading) of specific chemical variables (e.g., amino acids, fatty acids, minerals) to each component. Variables positioned far from the origin along an axis significantly influence that PC and correlate with the sample groupings seen in Panel A in that direction. Bar charts visualize the magnitude and direction (positive or negative) of these key variable loadings for interpreting the patterns observed in the score plot.

To explore the underlying biochemical variation among 60 safflower accessions, we performed principal component analysis (PCA), which revealed pronounced geography-associated chemodiversity in the germplasm collection^26,27^. Biochemical segregation was primarily described by the first four principal components (PCs), which together explained approximately 51% of the total variance (Fig. 2, Supplementary Table 3). The spatial distribution in the score plot revealed a ​​diagnostic biogeographical divergence​​: Chinese accessions (particularly ​​Xinjiang materials​​) exhibited strong aggregation along the ​​positive PC1 axis​​, forming a distinct chemotype characterized. In stark contrast, Near Eastern accessions (notably those from Syria) were distributed toward the negative PC1 axis. This east–west separation reflects distinct biochemical compositions rather than discrete genetic lineages. The observation that Near Eastern accessions appeared to be relatively richer in secondary metabolites was derived from loading values on PC1, where phenolic and flavonoid compounds contributed most strongly to the negative axis. These results indicate that environmental adaptation and cultivation history may have jointly shaped regional biochemical differentiation patterns in safflower germplasm. The geographic clustering revealed by PCA parallels the regional trends observed in the phenotypic clustering analysis (Fig. 1), suggesting shared evolutionary drivers across genetic and metabolic architectures^28^.

Critically, interrogation of PC1 loadings (Fig. 2B, Supplementary Table 3) identified ​​specific biochemical drivers​​ of this divergence: the amino acids ​​glutamate (Glu)​​ and ​​aspartate (Asp)​​ showed the strongest positive contributions, alongside the alkali earth minerals ​​magnesium (Mg)​​ and ​​calcium (Ca)​​. This co-enrichment indicates ​​adaptive cation-amino acid complexation​​, a biochemical strategy for maintaining ion homeostasis in the ​​alkaline soils predominant in Northwest China’s safflower-growing regions. Complementary ​​PC2 partitioning​​ reflected fundamentally distinct metabolic priorities: ω−3/ω−6 polyunsaturated fatty acids loaded negatively against terpenoid precursors, implying ​​environmentally modulated tradeoffs in lipid investment​​—likely mediating membrane fluidity adaptation to temperature gradients across sampling sites^29^. PC3 (7.7% variance)​​ exposed antagonistic loading between ​​crude fiber and the micronutrient ​​zinc (Zn)​​, indicative of cell wall-nutrient competition for carbon skeletons. Simultaneously, ​​PC4 (5.7% variance)​​ highlighted competitive accumulation between ​​iron (Fe)​​ and ​​γ-linolenic acid—evidence supporting redox avoidance through metabolic resource partitioning. Cumulatively, PCs 1–4 resolved ​​51% of total chemodiversity​​, establishing that geographic origin embeds a quantifiable biochemical signature governed by three evolutionary strategies: Ionic resilience (PC1)​​ via mineral-amino acid complexes for osmotic balance; Membrane plasticity control (PC2)​​ through lipid class specialization; Nutrient tradeoff management (PC3/PC4)​​ minimizing oxidative conflict; This layered architecture demonstrates how ​​spatial isolation drives nested metabolic adaptation​​ in safflower, with dominant principal components (PC1/PC2) resolving continental-scale divergence, while subordinate axes (PC3/PC4) decode local-scale biochemical negotiations. Although the first four principal components explain 51% of total variance, this level is typical for high-dimensional biochemical datasets, where numerous correlated traits contribute to smaller incremental variance per component. The observed clustering patterns therefore remain biologically meaningful and interpretable.

### Nutrient interaction networks highlight Mineral-Fatty acid Antagonism​​

**Fig. 3:**
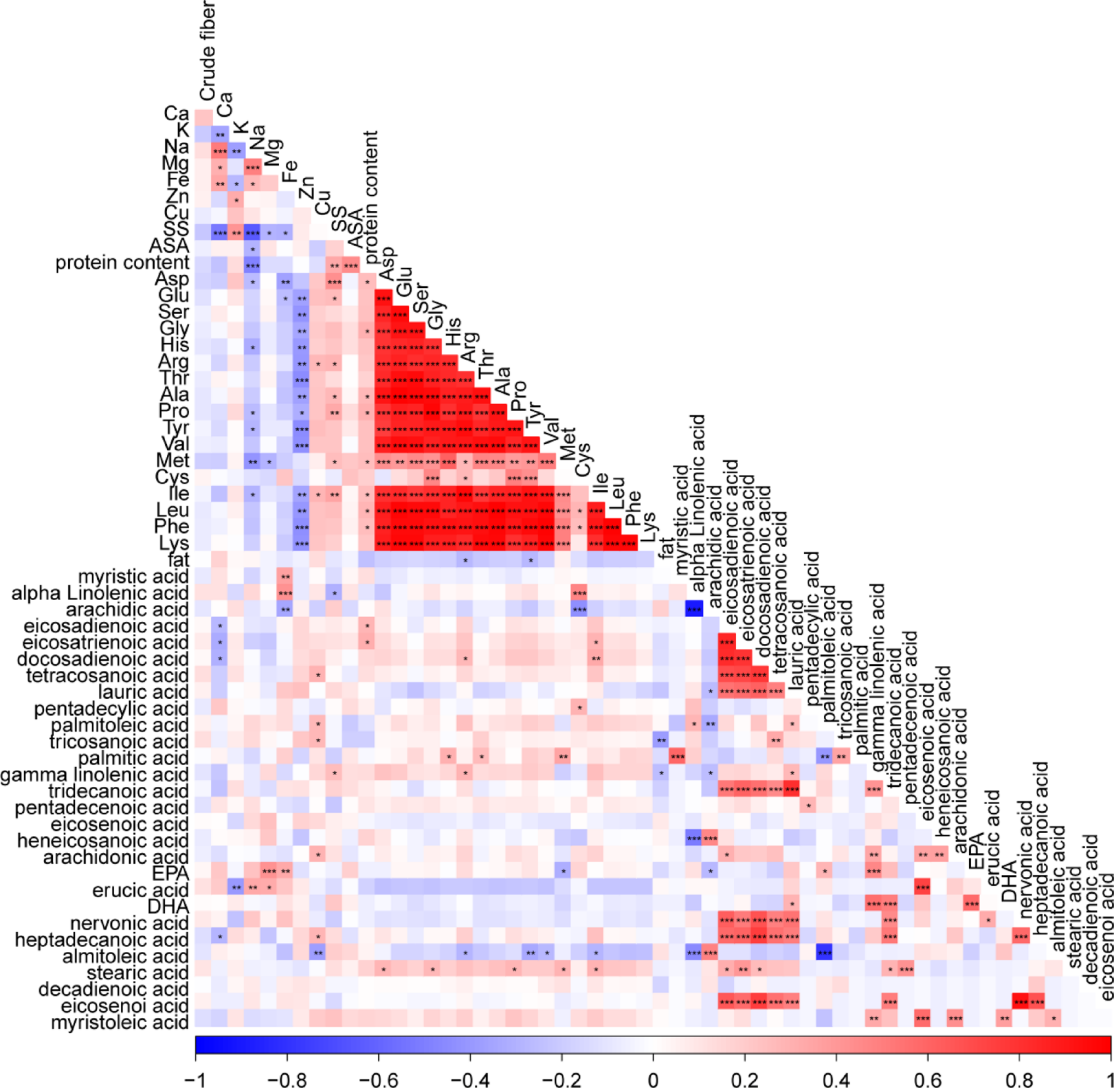
Correlation heatmap of safflower chemical variables. Heatmap of Pearson correlations between 57 biochemical traits: crude fiber, minerals (Ca, K, Na, Mg, Fe, Zn, Cu), protein metrics (protein and 17 amino acids), SS, ASA, and fatty acids (e.g., myristic acid, DHA, EPA). Color scale: red (r = + 1), blue (*r* = − 1). Key patterns: (i) Positive mineral-mineral correlations (red blocks, e.g., Ca-K), (ii) Amino acid co-expression (e.g., Asp-Lys cluster), (iii) Negative mineral-fatty acid relationships (blue squares, e.g., Fe vs. palmitic acid). Asterisks denote significance levels: ^*^ *p* < 0.05, ^**^ *p* < 0.01, and ^***^ *p* < 0.001.

To further explore the relationship between samples, we analyzed the correlation network of 57 biochemical traits, which reveals systemic mineral-fatty acid antagonism as a defining feature of safflower metabolism (Fig. 3, Supplementary Table 4). Our correlation matrix exposes ​​a three-tiered hierarchical organization​​: cationic minerals (K, Ca, Mg) form intense co-accumulation hubs within the mineral quadrant (top-left 6 × 6 block), exhibiting positive correlations. This mineral solidarity starkly contrasts with their broad-spectrum inhibition of polyunsaturated fatty acids (PUFAs), manifesting as ​​pervasive blue sectors​​ across the mineral-fatty acid interface^30^. The most pronounced antagonisms occur in the transition metal-PUFA submatrix: Fe establishes profound negative correlations with C18:3 ω−3 fatty acids (α-linolenate, γ-linolenate​​), while Zn similarly antagonizes EPA and DHA^31^. Crucially, this redox-mediated conflict peaks at Fe/γ-linolenate, the matrix’s strongest inverse relationship, which indicates selective suppression of peroxidation-prone lipids near iron accumulation zones^24^.

Amino acids occupied a pivotal position in the correlation network, mediating interactions between mineral nutrients and fatty acids. Acidic amino acids such as aspartic acid (Asp) and glutamic acid (Glu) exhibited dual-gradient relationships—positively correlated with mineral cations (e.g., Ca and Mg; salmon-pink clusters) but negatively correlated with long-chain polyunsaturated fatty acids (PUFAs; C20+)^32,33^. In contrast, hydrophobic amino acids (Leu, Ile, Val) displayed lipid-aligned patterns, correlating positively with saturated fatty acids such as palmitic acid while showing minimal association with mineral ions. This partitioning reflects distinct biosynthetic and physiological strategies: proteinogenic amino acids facilitate mineral homeostasis and osmotic balance, whereas fatty acids contribute to membrane structural adaptation^19,21^. Collectively, the nutrient network reveals a tripartite organization in which minerals, amino acids, and lipids occupy separate but interconnected metabolic domains. The observed mineral–fatty acid antagonism, bridged by amino acid interactions, likely represents a biochemical coordination mechanism that stabilizes safflower cellular metabolism under environmental stress^31^. This architecture suggests an adaptive trade-off between membrane fluidity (driven by PUFAs) and ionic equilibrium (maintained by minerals), defining the species’ resource-allocation framework for survival in variable agroecological settings^34^.

### Machine learning models enable prediction of fiber content

**Fig. 4:**
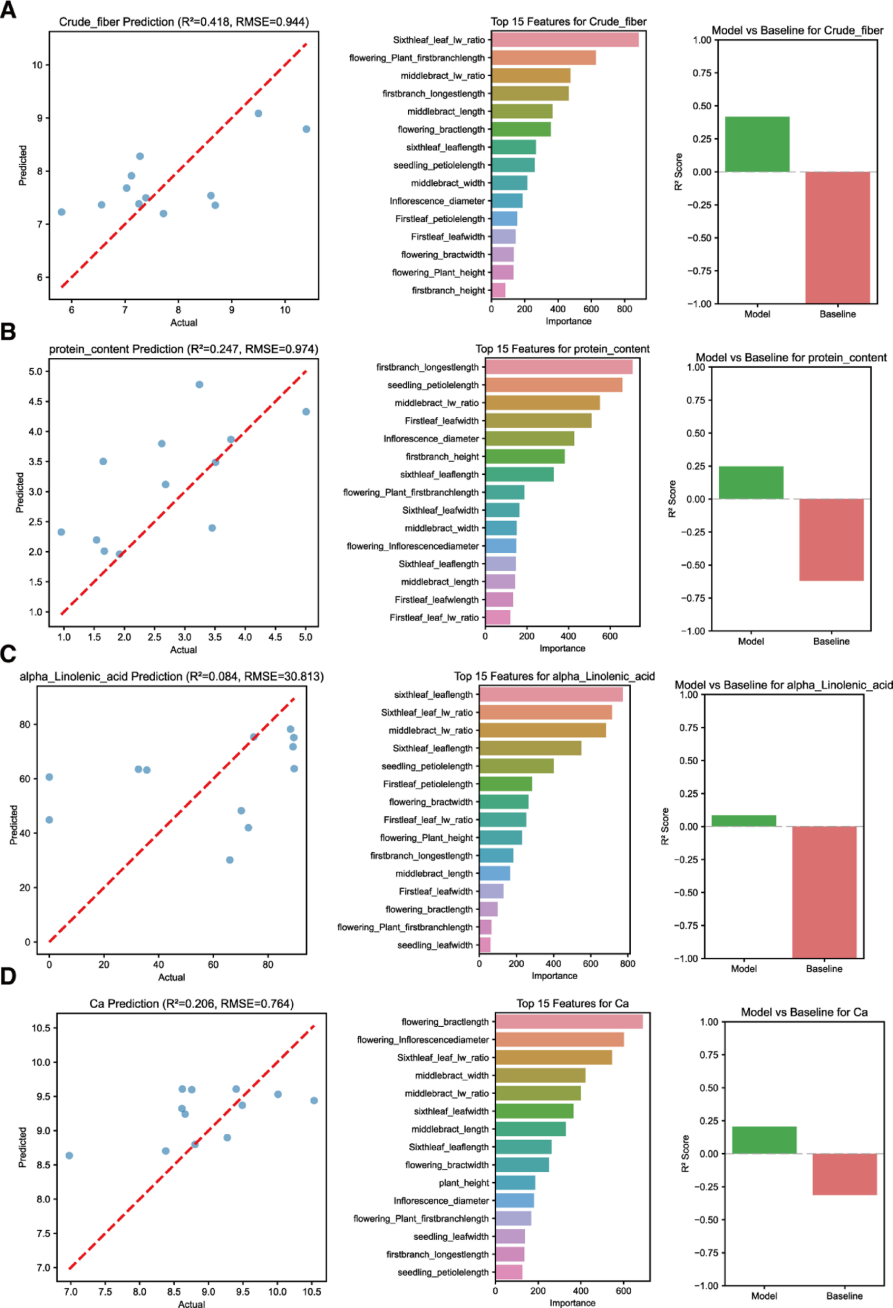
Predictive modeling of safflower nutritional traits and key feature importance.​​ Composite panel **(A–D)** evaluate machine learning model performance and compare predictive power against baseline models, highlighting biochemical drivers for four nutritional targets. The left column shows actual vs. predicted scatter plots for each trait, and the middle bar charts displays the top 15 most important features. The right column compare model R² scores with baseline performance. **(A)** Crude fiber prediction (R² = 0.418, RMSE = 0.944). **(B)** Protein content prediction (R² = 0.247, RMSE = 0.974). **(C)​​** α-Linolenic acid prediction (R² = 0.084, RMSE = 30.813). **(D)** Calcium (Ca) prediction (R² = 0.206, RMSE = 0.764).

To elucidate the biological foundations underlying safflower trait predictability^7^, we employed machine learning to decode the hierarchical regulation of key phenotypic and biochemical traits (Fig. 4, Supplementary Table 5). Our analysis revealed that crude fiber content (R² = 0.418, RMSE = 0.944) exhibits relatively higher predictability compared to other traits, such as protein (R² = 0.247, RMSE = 0.974), α-linolenic acid (R² = 0.084, RMSE = 30.813), and calcium (R² = 0.206, RMSE = 0.764), with this performance disparity rooted in fundamental differences in biological organization. The relatively modest R² values across all traits partly reflect limitations in sample size (*n* = 60 accessions), which constrained the model ability to capture the full spectrum of genetic and environmental variability, particularly for highly plastic traits like fatty acids and minerals^19,21^. However, comparison against baseline models that predicted randomized or mean trait values confirmed that the observed R² values exceeded those expected by chance, underscoring the true predictive power of the gradient-boosted models rather than artifacts of model fitting. The close correspondence between predicted and observed fiber values, absent in more metabolically labile traits, which suggests that fiber accumulation follows a more stable developmental program. SHAP analysis demonstrated that morpho-developmental features like flowering plant length dominate fiber prediction, while nutrient-related factors contribute minimally (< 8%), indicating that fiber deposition is primarily governed by structural determinants rather than metabolic competition. This mechanistic distinction explains why fiber, despite being a quantitative trait, proves more computationally tractable than nutritionally contested compounds, its biosynthesis appears evolutionarily hardwired to plant architecture through cellulose synthase (CesA) gene clusters that operate independently of the antagonistic mineral-PUFA networks constraining other traits. This is further evidenced by fiber’s moderate coefficient of variation (CV = 22.58%) compared to the extreme variability of fatty acids (CV > 130%).

These findings fundamentally reshape our understanding of safflower trait regulation, demonstrating that predictive accuracy is determined not by trait complexity, but by the degree of metabolic insulation from competing biosynthetic pathways. The superior model performance for fiber content establishes it as a “quantitative trait island”, a characteristic uniquely amenable to genetic improvement due to its developmental canalization and freedom from resource allocation tradeoffs. Our work reveals how evolutionary constraints shape trait predictability across different biochemical classes. We concluded that crude fiber as a high-priority target for marker-assisted selection in safflower breeding programs, and it offers a framework for identifying similarly tractable traits in other medicinal crops. By demonstrating that structural traits can be more predictable than nutritional qualities despite their polygenic nature, our study opens new avenues for precision breeding strategies that leverage the modularity of plant metabolic networks, with significant implications for improving the yield and quality of safflower and related medicinal species in the face of climate change and growing global demand for plant-based therapeutics.

## Discussion

Our analyses reveal that ​​geographical isolation drives convergent evolution of nutrient signatures​​ in safflower. The extreme genetic bottleneck observed in Xinjiang accessions correlates tightly with their distinctive biochemical profile, particularly mineral-amino acid complexes dominating PC1 (26% variance). This suggests adaptive selection for ​​cationic mineral homeostasis​​ (Mg, Ca) as an evolutionary response to alkaline soils in arid Northwest China^35^. Crucially, the biochemical divergence between Near Eastern (Syrian) and East Asian germplasm mirrors continental-scale genetic structuring, indicating that ​​soil geochemistry exerts stronger selective pressure than climate​​ on nutrient allocation^34^. Our findings align with recent studies in Carthamus species demonstrating metalloenzyme adaptations to high-pH environments but extend the paradigm by showing how geographical constraints amplify nutrient signature conservation through reduced gene flow^23^.

The antagonistic mineral-fatty acid networks uncovered in our study expose ​​fundamental resource allocation tradeoffs​​ governing safflower metabolism. We propose this reflects an evolutionary strategy to ​​minimize oxidative stress​​: lipid peroxidation risk from ω−3/ω−6 accumulation is mitigated by suppressing transition metal abundance (Fe, Cu), a mechanism potentially conserved across Asteraceae^19,31^. Our machine learning models further quantify these constraints: crude fiber’s high predictability (R² = 0.418) stems from its decoupling from contested nutrient pools, whereas minerals lower predictability arises from their integration with amino acid metabolism. This explains why fatty acids with extreme CVs remain evolutionarily stable, their biosynthesis operates in metabolic “silos” buffered from mineral antagonism. Importantly, these tradeoffs limit simultaneous optimization of nutraceutical traits, demanding strategic breeding approaches.

We reveal non-intuitive regulatory hierarchies via LightGBM-SHAP framework. For instance, amino acids outperforming minerals as calcium predictors (Fig. 4D) implies ​​amino acid-mediated transport​​ dominates over passive accumulation, a hypothesis testable through transporter knockout studies^6^. Furthermore, the prominence of developmental features in fiber prediction suggests ​​structural carbohydrate allocation is gated by phenology, explaining why conventional nutrient manipulations often fail to enhance fiber yield^20,23,28^. Our identification of high-GDI traits provides immediate targets for marker-assisted selection, while the geographical fingerprinting model enables provenance authentication, critical for safeguarding medicinal safflower value chains. Future integration with genome-wide association studies could resolve whether observed metabolic tradeoffs stem from pleiotropic regulators^35^.

These advances would bridge our computational insights with practical precision breeding, positioning safflower as a model system for studying evolutionary constraints in nutraceutical crop optimization​​, particularly relevant given climate-driven expansion of marginal agricultural lands.

## Limitations of the study

While this study provides novel insights into safflower’s geographical nutrient signatures and metabolic tradeoffs, several limitations warrant consideration. ​​Sample size constraints​​ (*n* = 60 accessions) limit the statistical power for detecting subtle genotype-environment interactions, particularly for traits with extreme variability. The ​​geographical scope​​, though diverse, underrepresents key safflower-growing regions, potentially biasing the identified Xinjiang bottleneck effect and reducing global generalizability^3^. ​​Machine learning limitations​​ include dependency on feature engineering and an inability to fully resolve causal mechanisms behind mineral-fatty acid antagonisms, despite high predictive performance for crude fiber (R² = 0.418). ​​Data heterogeneity​​—stemming from multi-origin field samples—introduced uncontrolled environmental noise, which preprocessing mitigated but could not eliminate^6^. We acknowledge that our machine-learning framework adopts a simplified configuration that does not include full-scale hyperparameter optimization or nested cross-validation. This was a deliberate choice given the limited sample size, as more complex procedures would likely overfit and reduce reproducibility. Finally, the ​​lack of multi-omics validation​​ leaves the proposed evolutionary tradeoffs as hypotheses awaiting experimental confirmation. Future studies should prioritize expanded germplasm panels with controlled phenotyping and SHAP-driven mechanistic validation to address these gaps^20,28,36^.

## Conclusion

Our study reveals safflower biochemical architecture through machine learning-driven analysis, establishing that ​​geographical origin encodes diagnostic nutrient signatures​​ via genetically constrained metabolic tradeoffs. We demonstrated that Xinjiang germplasm exhibits unique biochemical homogenization, quantified by phylogenetic bottlenecks and PCA-driven chemodiversity, where cationic mineral-amino acid complexes optimize ion homeostasis in alkaline soils. Critically, our LightGBM models exposed ​​fundamental biosynthetic constraints​​: crude fiber’s high predictability reflects developmental hardwiring with plant architecture, while mineral-fatty acid antagonisms limit micronutrient-PUFA co-optimization. We revealed a brand new safflower evolutionary metabolic strategy, ​​resource partitioning between structural carbohydrates and redox-sensitive lipids​​, while providing actionable targets: high-GDI traits serve as priority selection markers for breeding programs. Our future efforts will expand this framework to integrate genome-wide associations, enabling predictive breeding for climate-resilient safflower cultivars with enhanced nutraceutical value.

## Materials and methods

### Sample collection and Preparation

Our research safflower materials of 60 accessions were obtained from the *National Central Asian Characteristic Crop Germplasm Resources Medium-term Gene Bank (Urumqi*,* China)*. These accessions represent diverse agroecological origins across provinces of China and foreign accessions from Syria, Australia, Mexico, Japan, India, and the U.S. This design ensured that multiple production regions and eco-types were represented, thereby minimizing potential sampling bias. To reduce environmental effects, all accessions were propagated under standardized field conditions at the same experimental station, with identical soil type, irrigation, and light management. After flowering and maturation, plant tissues including florets and seeds were harvested at consistent phenological stages. Seed samples were air-dried and milled to a fine powder using a 60-mesh grinder. Powdered materials were stored at − 80 °C until further biochemical analysis. Floral tissues were immediately flash-frozen in liquid nitrogen and stored for metabolomic profiling. All biochemical assays, including crude fiber, protein, mineral content, and fatty acid composition, which were performed in biological triplicates, with quality control standards and inter-assay calibration to ensure data reproducibility.

### Phenotypic trait measurement

We evaluated safflower phenotypic traits by the technical protocols outlined in the *Guidelines for the Conduct of Tests for Distinctness*,* Uniformity and Stability – Safflower* (NY/T 2753–2015). For individual trait measurements, we randomly selected at least 20 plants per accession. We collected one representative organ from each plant when assessing organ-specific traits. For population-level observations, we recorded data based on either the entire plot or a predefined bulked sample representative of the population. We documented traits such as plant height, branch number, head diameter, flowering time, and seed color using standardized descriptors. Trait variability was quantified using the coefficient of variation (CV) and genetic diversity index (GDI). For each trait, the minimum (min), maximum (max), mean, and standard deviation (SD) were calculated across all accessions. Trait stability was assessed using the coefficient of variation (CV), where CV < 11% indicates high stability, 11–20% moderate stability, and > 20% low stability^37^. The genetic diversity index (GDI) was interpreted in a relative sense, with higher values denoting greater phenotypic diversity among accessions. All trait values were experimentally determined in this study from biochemical assays, rather than sourced from any external database or previous publication. All measurements were conducted across triplicate plots under consistent cultivation and management conditions to minimize environmental variation and enhance data reliability.

### Phylogeographic segmentation

To infer phenotypic relationships among safflower accessions, a hierarchical clustering–based phylogenetic analysis was performed using standardized trait data. A total of 57 biochemical traits were used to construct a phenotypic similarity matrix. Data clustering and visualization were conducted with default parameters in R v4.3.2.

### ​​Soluble sugar quantification​​

Soluble sugar (SS) content in safflower tissues was determined colorimetrically using an anthrone-sulfuric acid assay kit (G0501W, Grace Biotechnology)^38,39^. Fresh tissue was extracted in 1.5 mL 80% ethanol at 50 °C for 20 min, followed by centrifugation. Supernatants were reacted with anthrone reagent and concentrated sulfuric acid at 95–100 °C for 10 min. Absorbance was measured at 620 nm using a microplate reader. SS concentrations were calculated against a glucose standard curve and normalized to fresh weight using the formula:

$$\:\text{S}\text{S}=\:0.718\:\times\:\:\left({\Delta\:}\text{A}\:+\:0.0103\right)\times\:\:\frac{D}{W}\text{}\text{}$$

where ΔA = sample absorbance minus blank, D = optimized dilution factor, and W = sample weight (g).

### Crude fat determination

We quantified the crude fat content in safflower seeds following the national standard protocol outlined in *Determination of Oil Content in Oilseeds* (GB/T 14488.1–2008). We first air-dried the seed samples and ground them into a fine powder using a 60-mesh sieve. We then accurately weighed approximately 2–3 g of the powdered sample and subjected it to Soxhlet extraction using petroleum ether as the solvent. The extraction was carried out under controlled temperature and reflux conditions for a minimum of 6 h. After extraction, we evaporated the solvent and dried the remaining residue to a constant weight to determine the crude fat content. We performed all measurements in triplicate to ensure analytical consistency.

### Fatty acid profiling

Fatty acid composition was determined by *National Food Safety Standard—Determination of Fatty Acids in Foods* (GB 5009.168–2016). Safflower seed powder was subjected to acid-catalyzed methylation to convert fatty acids into their methyl esters (FAMEs). The derivatized FAMEs were analyzed using gas chromatography (GC) equipped with a flame ionization detector (FID) and a capillary column suitable for fatty acid separation. Identification and quantification of fatty acids were performed by comparing retention times with known standards. Each sample was analyzed in triplicate, and fatty acid content was expressed as a percentage of total fat.

### ​Micronutrient analysis

Quantification of calcium (Ca), potassium (K), sodium (Na), magnesium (Mg), iron (Fe), zinc (Zn), and copper (Cu) in safflower samples followed established protocols for plant mineral analysis. Fresh leaf tissues were oven-dried, finely ground (< 40-mesh sieve), and homogenized before digestion. Samples underwent mixed-acid digestion using a graphite block digestion system (HaiNeng SH220N/LeCi KDNX-20) with concentrated nitric acid and hydrogen peroxide. Elemental concentrations were determined via flame atomic absorption spectrometry (FAAS) using a Puxi TAS-990G instrument with deuterium background correction.

### Ascorbic acid quantification​​

Reduced ascorbic acid (AsA) content in safflower tissues was determined using a colorimetric iron reduction assay (Reduced Ascorbic Acid Assay Kit, G0201W, Grace Biotechnology). Fresh tissue was homogenized in 1 mL ice-cold extraction buffer, centrifuged, and the supernatant collected. For detection, 200 µL supernatant was mixed sequentially with assay reagents, incubated at 30 °C for 60 min, and centrifuged if precipitate formed. Absorbance of the supernatant was measured at 534 nm.

### Protein content quantification

Protein content was quantified using the Coomassie Brilliant Blue (Bradford) assay. Approximately 0.1 g of fresh tissue was homogenized in ice-cold extraction buffer, followed by centrifugation. The supernatant was collected and typically diluted 10-fold to ensure readings fell within the linear range. Protein concentration was determined by mixing diluted sample supernatant with Coomassie reagent. After incubating, absorbance was measured at 600 nm using a microplate reader, with distilled water serving as the blank.

### Amino acid profiling

For the quantification of 17 amino acids in safflower, samples were cryogenically homogenized in liquid nitrogen, and approximately 0.2 g of the finely ground material underwent acid hydrolysis with 1.5 mL of 6 mol/L HCl at 110 °C for 24 h under nitrogen atmosphere. The hydrolysate was centrifuged, and the supernatant was collected, vacuum-dried to remove residual acid, and reconstituted in ultrapure water. Before chromatographic analysis, the sample extract underwent pre-column derivatization: addition of 20 µL norleucine was followed by reaction with 100 µL triethylamine and 100 µL phenylisothiocyanate (PITC) at room temperature for 1 h. Excess reagent was removed by liquid-liquid extraction with 400 µL n-hexane. Amino acid derivatives were separated and quantified using reversed-phase high-performance liquid chromatography (HPLC) on a dedicated amino acid column using a binary gradient system at 1.0 mL/min, 35 °C, with UV detection at 254 nm.

### Interaction network analysis

We constructed a correlation-based nutrient interaction network encompassing 57 biochemical variables, including crude fiber, minerals, protein and amino acid profiles, soluble sugars, antioxidant activity, and fatty acids. Pairwise Pearson correlation coefficients and corresponding *p*-values were computed using the Hmisc package in R v4.3.2. Visualization of correlation structures was performed with the corrplot package in R v4.3.2.

### ​Machine learning framework for predictive trait modeling

To establish predictive models for key safflower biochemical traits, we implemented an integrated computational pipeline leveraging LightGBM gradient boosting. Given the limited sample size, extensive hyperparameter tuning or complex model selection procedures would risk severe overfitting. Therefore, we adopted a restrained and stable parameter configuration to ensure reproducibility and prevent overfitting. Model performance was quantified by the coefficient of determination (R²) and root mean square error (RMSE). To assess the added predictive value of the machine learning models, results were compared against a baseline model that predicted the mean trait value from the training data (mean regression baseline). Biological interpretability was further enhanced through SHAP-based feature importance analysis and partial dependence plots to elucidate trait–predictor relationships. All computations were executed in Python 3.9 using scikit-learn and LightGBM libraries.

## Supplementary Information

Below is the link to the electronic supplementary material.

Supplementary Material 1

## Data Availability

Any additional information required to reanalyze the data reported in this paper is available from the lead contact upon request. All codes used for data analysis, visualization, and machine learning are available at the GitHub: [https://github.com/Zlj4537163/ML_Zhao.2025](https:/github.com/Zlj4537163/ML_Zhao.2025).
